# Supplementary material for: Antifungal Activities of Bacillus mojavensis BQ-33 towards the Kiwifruit Black Spot Disease Caused by the Fungal Pathogen Didymella glomerata
Source: Microorganisms. 2022 Oct 21;10(10):2085. doi: 10.3390/microorganisms10102085 (PMC9611226; doi:10.3390/microorganisms10102085)
Supplement: Supplementary file 1 [file microorganisms-10-02085-s001.zip › microorganisms-1974276-supplementary.pdf]

Antifungal activities of *Bacillus mojavensis* BQ-33 towards the kiwifruit black spot disease caused by the fungal pathogen *Didymella glomerata*

Table S1. Antagonistic evaluation of bacterial strains against *Didymella glomerata*

Stran's name	Sources	Inhibition ratio
BQ-4	soil Guizhou	57.70 ± 0.65 opq
BQ-5	soil Guizhou	60.37 ± 0.73 mn
BQ-7	soil Guizhou	63.99 ± 1.46 ijk
BQ-9	soil Guizhou	65.80 ± 0.72 hi
BQ-10	soil Guizhou	59.63 ± 0.69 mno
BQ-11	soil Guizhou	65.56 ± 0.46 hi
BQ-13	soil Guizhou	61.29 ± 0.83 lm
BQ-16	soil Guizhou	77.15 ± 0.54 b
BQ-18	soil Guizhou	71.52 ± 0.47 f
BQ-19	soil Guizhou	55.46 ± 0.71 qr
BQ-21	soil Guizhou	64.27 ± 0.93 ij
BQ-23	soil Guizhou	55.63 ± 0.67 qr
BQ-24	soil Guizhou	73.40 ± 0.68 def
BQ-25	soil Guizhou	64.54 ± 0.69 ij
BQ-27	soil Guizhou	75.13 ± 0.48 bcd
BQ-28	soil Guizhou	74.38 ± 0.58 cde
BQ-29	soil Guizhou	74.15 ± 1.00 cde
BQ-31	soil Guizhou	57.11 ± 1.30 pq

BQ-32	soil Guizhou	65.45 ± 0.79 hi
BQ-33	soil Guizhou	81.26 ± 0.53 a
BQ-38	soil Guizhou	62.72 ± 0.80 jkl
BQ-41	soil Guizhou	54.61 ± 0.52 rs
BQ-42	soil Guizhou	51.48 ± 0.39 t
BQ-44	soil Guizhou	61.73 ± 0.91 klm
BQ-47	soil Guizhou	58.52 ± 0.46 nop
BQ-48	soil Guizhou	62.85 ± 0.42 jkl
BQ-50	soil Guizhou	60.80 ± 0.34 lm
BQ-53	soil Guizhou	53.80 ± 0.81 rs
BQ-54	soil Guizhou	64.69 ± 1.21 ij
BQ-58	soil Guizhou	67.27 ± 0.48 gh
BQ-64	soil Guizhou	56.12 ± 0.39 qr
BQ-66	soil Guizhou	69.24 ± 1.09 g
BQ-71	soil Guizhou	52.81 ± 0.36 st
BQ-73	soil Guizhou	72.42 ± 0.78 ef
BQ-77	soil Guizhou	76.27 ± 0.60 bc
BQ-78	soil Guizhou	68.07 ± 0.48 g

Note: Numerical values were expressed as mean ± standard error (SE) of triplicates. Different lowercase letters represent a significant difference ($p < 0.05$, n = 3).

Table S2. Reference isolates used in the present study and their GenBank accession numbers.

Species	Culture Accession	GenBank Accession	
		16S rDNA	*gyrA*
Bacillus amyloliquefaciens	DSM7 T	FN597644	FN597644
Bacillus atrophaeus	NRRL NRS 213 T	LSBB01000022	EU138654
Bacillus halotolerans	KKD1	CP054584	CP054584
Bacillus halotolerans	MBH1	CP070976	CP070976
Bacillus inaquosorum	NRRL B-23052 T	NR-116188	EU138605
Bacillus mojavensis	UCMB5075 T	CP051464	CP051464
Bacillus nakamurai	NRRL B-41091 T	NR151897	LSAZ00000000
Bacillus siamensis	KCTC 13613 T	AJVF01000001	AJVF01000001
Bacillus sonorensis	NRRL B-23154 T	MF446619	EU138611
Bacillus spizizenii	NRRL B-23049 T	NR024931	EU138602
Bacillus subtilis	NCIB3610 T	CP020102	EF134425
Bacillus subtilis	NRRL B-4219 T	NR116183	EU138592
Bacillus subtilis	TU-B-10 T	NC016047	NC016047
Bacillus vallismortis	NRRL B-14890 T	NR-116186	EU138601
Bacillus velezensis	SQR9 T	CP006890	CP006890
Bacillus velezensis	NRRL B-41580 T	LLZC00000000	EU138622
Bacillus velezensis	FZB42 T	NC009725	NC009725
Halobacillus halophilus	DSM 2266 T	NC017668	NC017668

Note: The order of the strains was alphabetical. T = type strain.
